# Supplementary material for: A New Generation of Ultrasmall Nanoparticles Inducing Sensitization to Irradiation and Copper Depletion to Overcome Radioresistant and Invasive Cancers
Source: Pharmaceutics. 2022 Apr 7;14(4):814. doi: 10.3390/pharmaceutics14040814 (PMC9024746; doi:10.3390/pharmaceutics14040814)
Supplement: Supplementary file 1 [file pharmaceutics-14-00814-s001.zip › pharmaceutics-1633198-supplementary.pdf]

pharmaceutics

MDPI

Supplementary Materials: A New Generation of Ultrasmall Nanoparticles Inducing Sensitization to Irradiation and Copper Depletion to Overcome Radioresistant and Invasive Cancers

Paul Rocchi, Delphine Brichart-Vernos, François Lux, Isabelle Morfin, Laurent David, Claire Rodriguez-Lafrasse, Olivier Tillement

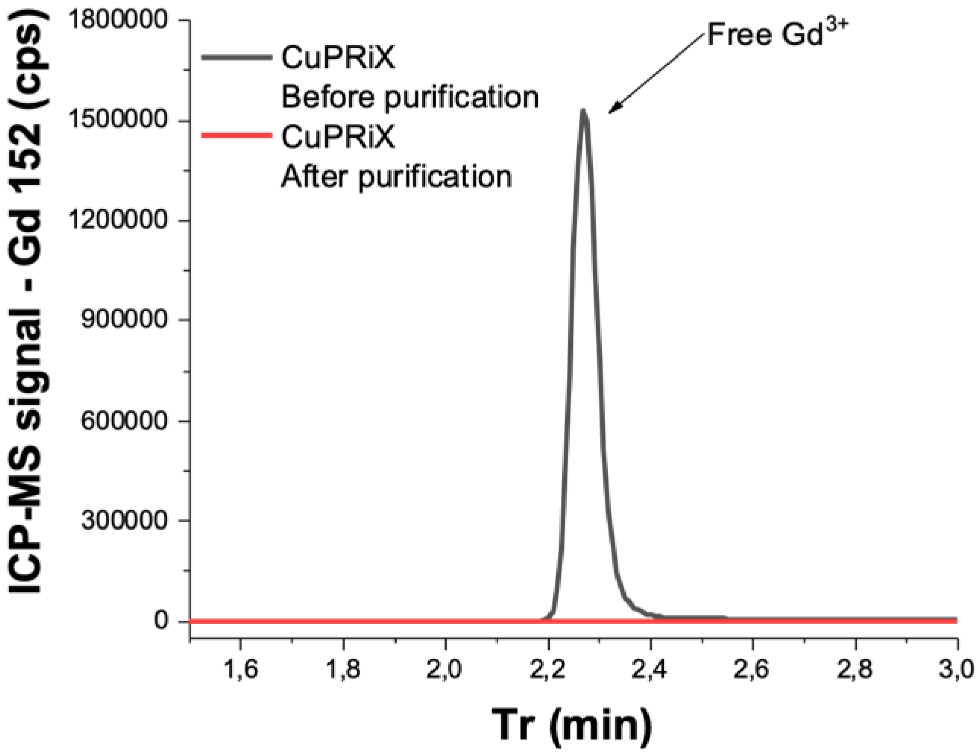

Figure S1. Comparison of free Gd^{3+} ions level before and after purification followed by HPLC-ICP/MS.

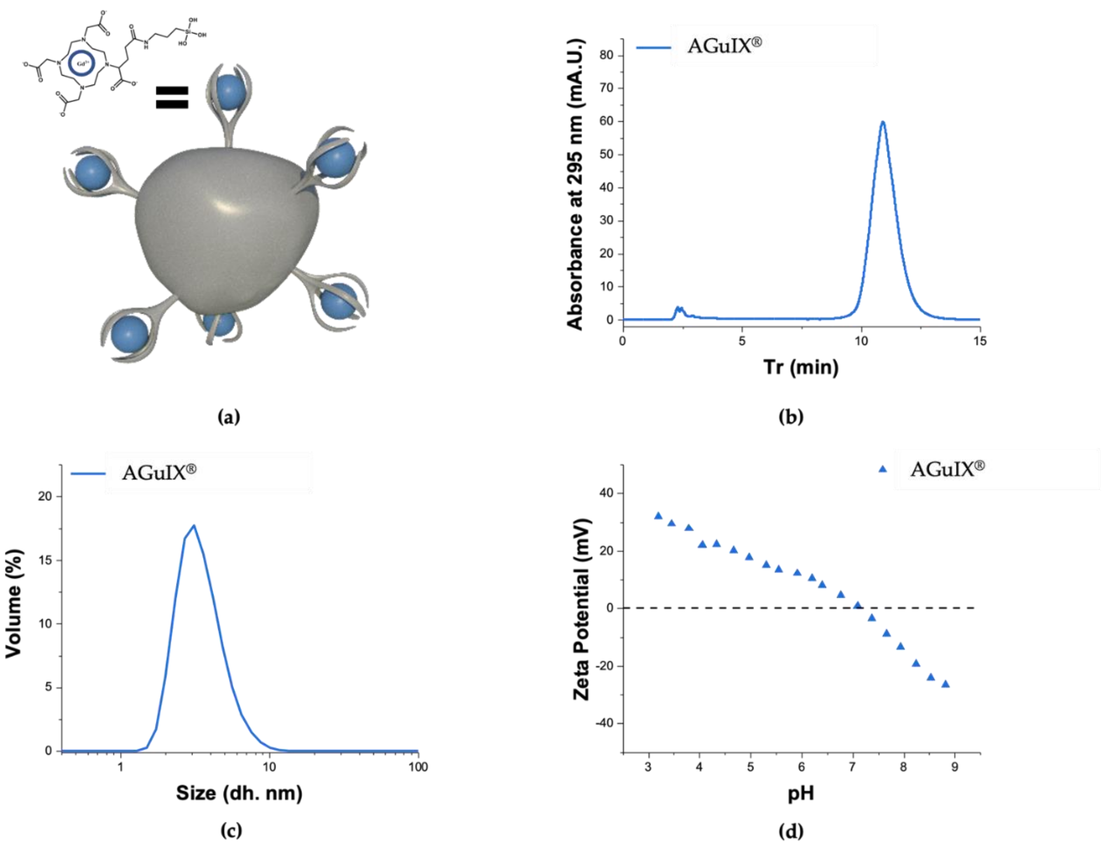

Figure S2. (a) Schematic representation of AGuIX® with the detailed structure of chelated DOTAGA groups; (b) HPLC-UV chromatograms of AGuIX® (10 µL, 100 g/L) recorded at 295 nm; (c) Hydrodynamic diameters distribution in volume as obtained by dynamic light scattering; (d) Zeta potential vs pH for AGuIX®.

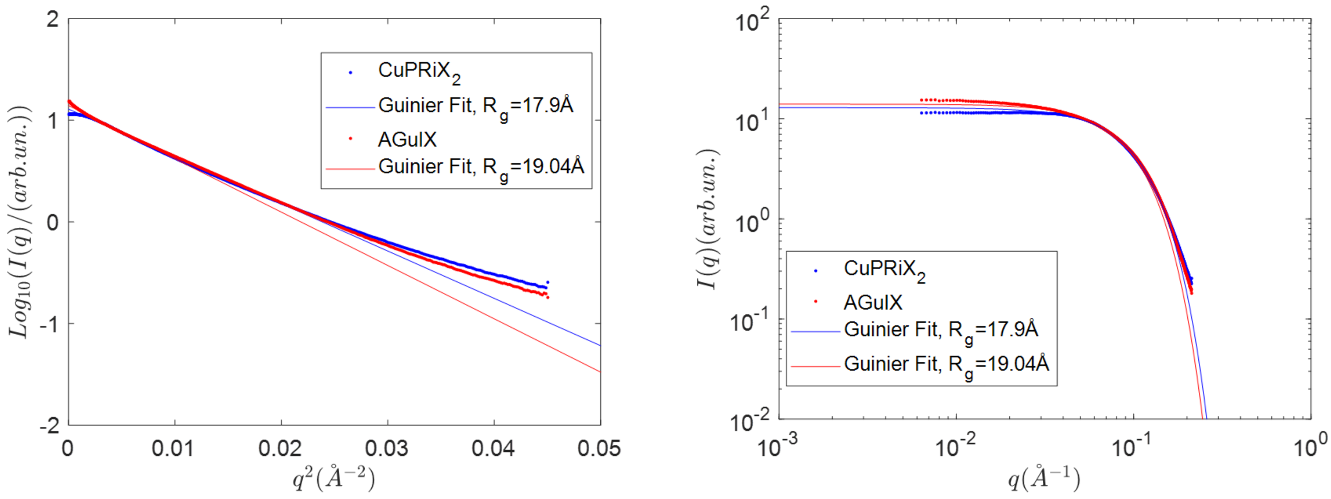

Figure S3. Calculation of radius of gyration by SAXS. (a) Guinier plots for $CuPRiX_2$ and AGuIX® hybrid nanoparticles. Both particles exhibit similar gyration radii. (b) Log-Log reprensentation using the same modelling as in (a), showing the deviation of the Guinier law in the high-q range ($q > 0.2$ Å^{-1}) and in the low-q range ($q < 3.10^{-3}$ Å^{-1}).

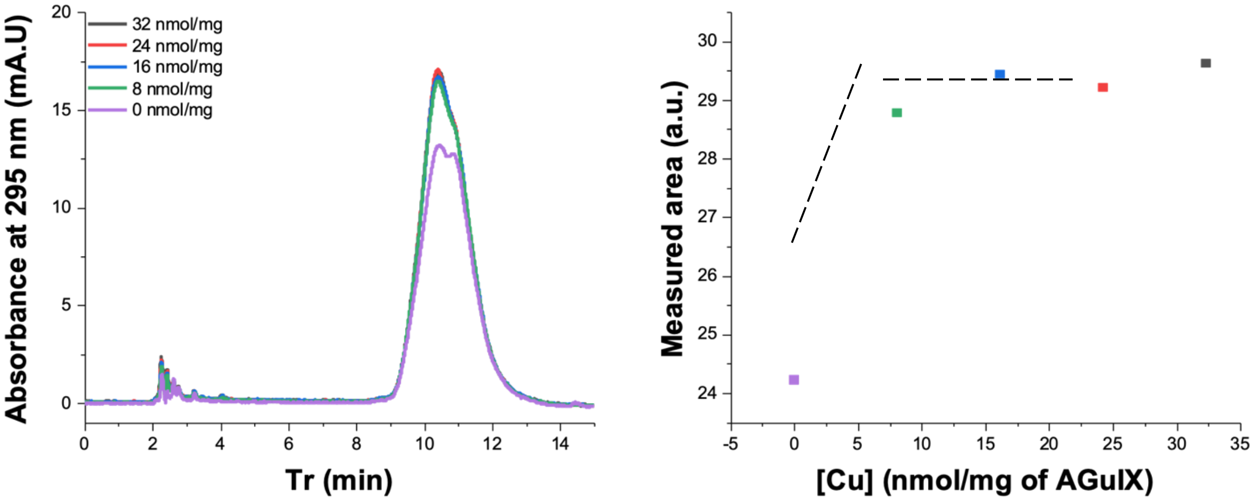

(a) (b)

Figure S4. Measurement of unchelated DOTAGA on AGuIX®. (a) Chromatograms of samples with increasing amount of Cu^{2+} per mg of AGuIX®. The increase in absorbance at 295 nm is due to the formation of DOTAGA@(Cu^{2+}); (b) Measured area depending on the amount of Cu^{2+}. The onset of the absorbance plateau shows the amount of DOTAGA on AGuIX®.

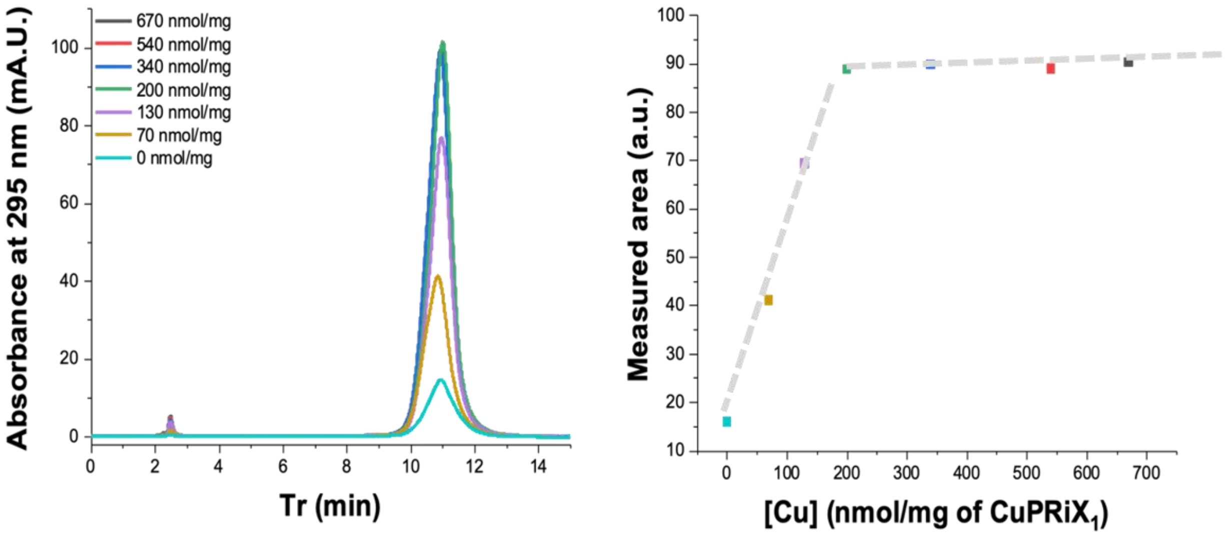

(a) (b)

Figure S5. Measurement of unchelated DOTAGA on CuPRiX$_1$. (a) Chromatograms of samples with increasing amount of Cu^{2+} per mg of CuPRiX$_1$. The increase in absorbance at 295 nm is due to the formation of DOTAGA@(Cu^{2+}); (b) Measured area depending on the amount of Cu^{2+}. The slope change shows the amount of DOTAGA on CuPRiX$_1$.

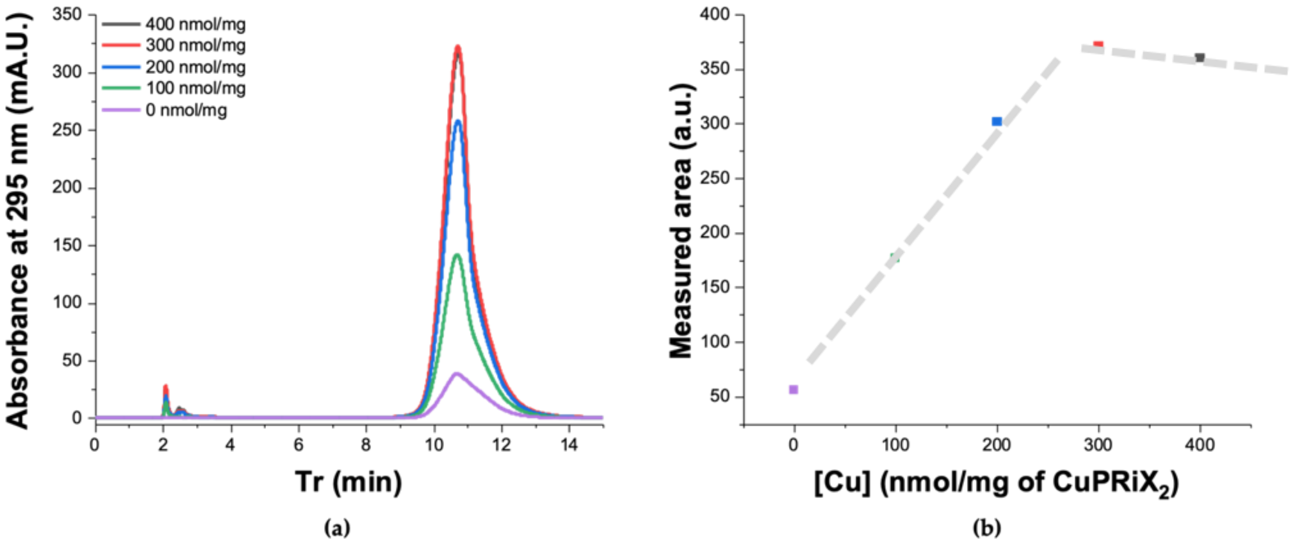

Figure S6. Measurement of unchelated DOTAGA on CuPRiX2. (a) Chromatograms of samples with increasing amount of Cu^{2+} per mg of CuPRiX2. The increase in absorbance at 295 nm is due to the formation of DOTAGA@(Cu^{2+}); (b) Measured area depending on the amount of Cu^{2+}. The slope change shows the amount of DOTAGA on CuPRiX2.

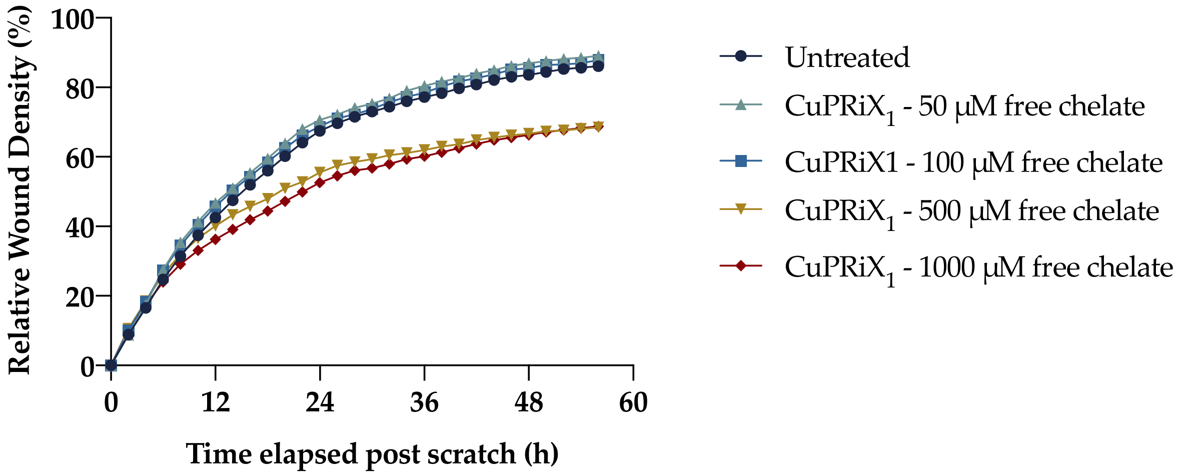

Figure S7. Effect of increasing concentrations of CuPRiX1 (0, 50, 100, 500, 1000 µM of uncomplexed chelate) on cell motility of A549 cells. Quantitative analysis of wound closure as a function of time. Relative wound density is a measure of the density of the wound region relative to the density of the cell region (%). Data are presented as the mean ± SEM (n=6).

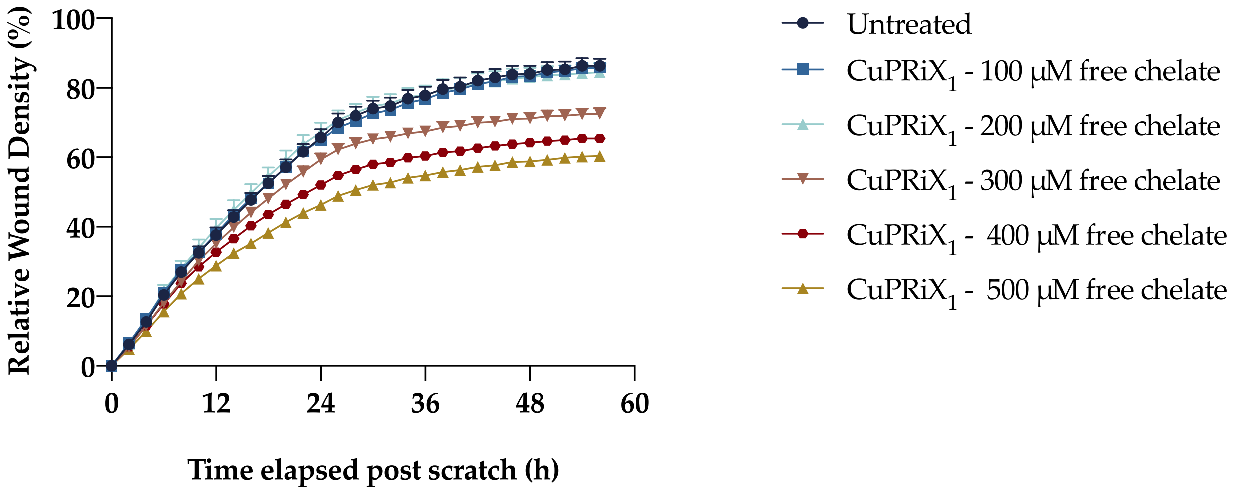

Figure S8. Effect of increasing concentrations of CuPRiX$_1$ (0, 100, 200, 300, 400, and 500 µM of uncomplexed chelate) on cell motility of A549 cells. Quantitative analysis of wound closure as a function of time. Relative wound density is a measure of the density of the wound region relative to the density of the cell region (%). Data are presented as the mean ± SEM (n=6).

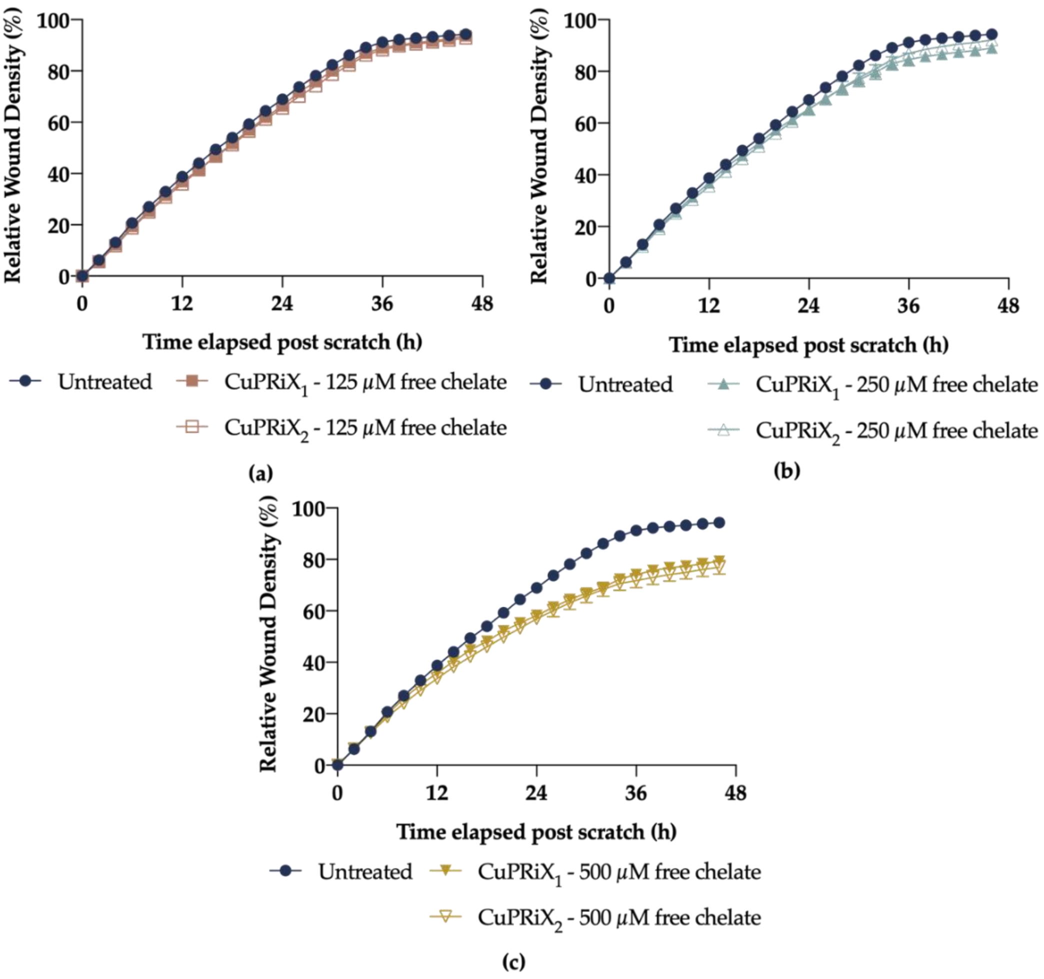

Figure S9. Effect of CuPRiX$_1$ and CuPRiX$_2$ on cell motility of A549 cells. (a) Treatment with 125 µM of uncomplexed chelate. (b) Treatment with 250 µM of uncomplexed chelate. (c) Treatment with 500 µM of uncomplexed chelate. Relative wound density is a measure of the density of the wound region relative to the density of the cell region (%). Data are presented as the mean ± SEM (n=6).

Proliferation Assay

A549 and SQ20B-CSCs cells were seeded in a 96-well plate at a density of 5,000 cells/well and incubated overnight at 37°C, 5% CO_2 to allow adhesion. Medium was removed and replaced with medium alone or containing $CuPRiX_1$ (500 µM of uncomplexed DOTAGA). The plates were placed in the IncuCyte ZOOM device and pictures were taken every 4 h for 48 h. Data were analyzed with the IncuCyte ZOOM software (v. 2018A), which allows quantification of cell surface coverage as confluence values.

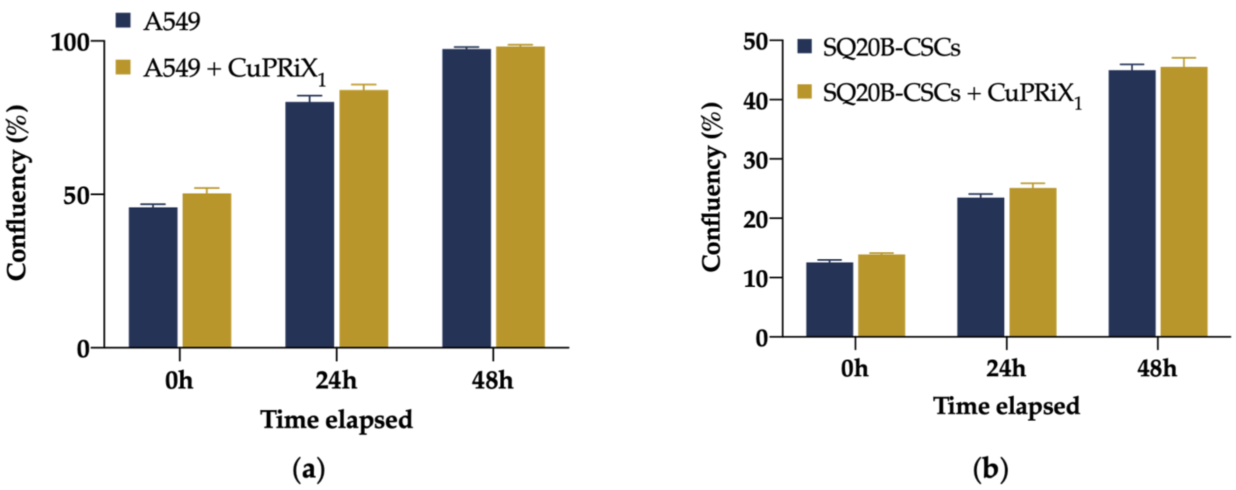

Figure S10. Proliferation of A549 (a) and SQ20B-CSCs (b) cells after treatment with $CuPRiX_1$ (500 µM of free DOTAGA). Cell proliferation was monitored with using IncuCyte ZOOM device, photos of the wells were taken every 6 hours for 48 h, and the percentage of cell confluency was expressed.
